# In Vivo Antiviral Effects of U18666A Against Type I Feline Infectious Peritonitis Virus

**DOI:** 10.3390/pathogens9010067

**Published:** 2020-01-18

**Authors:** Tomoyoshi Doki, Tomoyo Tarusawa, Tsutomu Hohdatsu, Tomomi Takano

**Affiliations:** Laboratory of Veterinary Infectious Disease, School of Veterinary Medicine, Kitasato University, Towada, Aomori 034-8628, Japan; doki@vmas.kitasato-u.ac.jp (T.D.); kitasato.denbyo+taru@gmail.com (T.T.); hohdatsu@vmas.kitasato-u.ac.jp (T.H.)

**Keywords:** feline coronavirus, feline infectious peritonitis, U18666A, cationic amphiphilic drug, antiviral agent

## Abstract

**Background:** The cationic amphiphilic drug U18666A inhibits the proliferation of type I FIPV in vitro. In this study, we evaluated the in vivo antiviral effects of U18666A by administering it to SPF cats challenged with type I FIPV. **Methods:** Ten SPF cats were randomly assigned to two experimental groups. FIPV KU-2 were inoculated intraperitoneally to cats. The control group was administered PBS, and the U18666A-treated group was administered U18666A subcutaneously at 2.5 mg/kg on day 0, and 1.25 mg/kg on days 2 and 4 after viral inoculation. **Results:** Two of the five control cats administered PBS alone developed FIP. Four of the five cats administered U18666A developed no signs of FIP. One cat that temporarily developed fever, had no other clinical symptoms, and no gross lesion was noted on an autopsy after the end of the experiment. The FIPV gene was detected intermittently in feces and saliva regardless of the development of FIP or administration of U18666A. **Conclusions:** When U18666A was administered to cats experimentally infected with type I FIPV, the development of FIP might be suppressed compared with the control group. However, the number of animals with FIP is too low to establish anti-viral effect of U18666A in cats.

## 1. Introduction

Coronaviruses are single-stranded positive-sense RNA viruses in the subfamily *Coronavirinae* of the family *Coronaviridae*. Coronaviruses have been classified into alpha-, beta-, gamma-, and deltacoronavirus [1]. Feline coronavirus (FCoV) belongs to species *Alphacoronavirus 1* of the genus *Alphacoronavirus*. *Alphacoronavirus 1* also includes porcine transmissible gastroenteritis virus (TGEV) and canine coronavirus (CCoV) [2,3].

The FCoV virion is mainly composed of nucleocapsid (N), envelope (E), membrane (M), and peplomer spike (S) proteins [4]. FCoVs are classified into two serotypes, type I and II FCoV, based on differences in the sequence of S protein and the 5′-region of the genome [5,6]. Type II FCoVs arise spontaneously by genomic recombinations between type I FCoV and type II CCoV [7,8,9]. Several serological and genetic surveys reported that type I FCoV is more prevalent than type II, therefore most cases of FIP are caused by type I FCoV infection [10,11,12].

FIP typically manifests effusion accumulation and granuloma formation. Ascites fluid is the most common effusion in cats with FIP, followed by pleural effusion. Granulomatous lesion is often observed on the surface of several organs, including the omentum, intestines, liver, kidneys, spleen, and lungs [13,14]. The mortality rate of cats that exhibit these symptoms is high. Recently, GS-441524 and GC-376 were developed as treatments for FIP. These drugs resolve the symptoms of FIP at a rate of 30%–80% [15,16,17,18,19]. They are expected to be used for the treatment of FIP. GS-441524 and GC-376 inhibit viral protein that is required for proliferation of FIPV. The escape virus for those drugs may appear by mutation on the viral protein. In fact, Pedersen et al. reported that one virus was resistant to GS-441524 in their field study [19]. Therefore, new drugs with different mechanisms of action from those drugs may be necessary.

U18666A is one of the cationic amphiphilic drugs (CADs) with cell permeability. It suppresses the function of the Niemann-Pick C1 protein (NPC1) of the cholesterol transporter and prevents the release of cholesterol from lysosomes [20]. U18666A was reported to have antiviral effects against dengue virus (DENV), hepatitis C virus (HCV), Zika virus (ZIKV), and chikungunya virus (CHIKV) by inhibiting the biosynthesis and intracellular transport of cholesterol [21,22,23,24]. We previously reported that U18666A also inhibits the intracellular transport of cholesterol and has high antiproliferative effects on type I FIPV at non-cytotoxic concentrations in the feline cell line [25]. Based on these results, U18666A is promising as an antiviral agent against type I FIPV. However, whether it also has antiviral effects on type I FIPV in vivo is unknown.

In this study, we evaluated the in vivo antiviral effects of U18666A by administering it to SPF cats challenged with type I FIPV.

## 2. Results

### 2.1. Experimental Schedule

**Figure 1:**
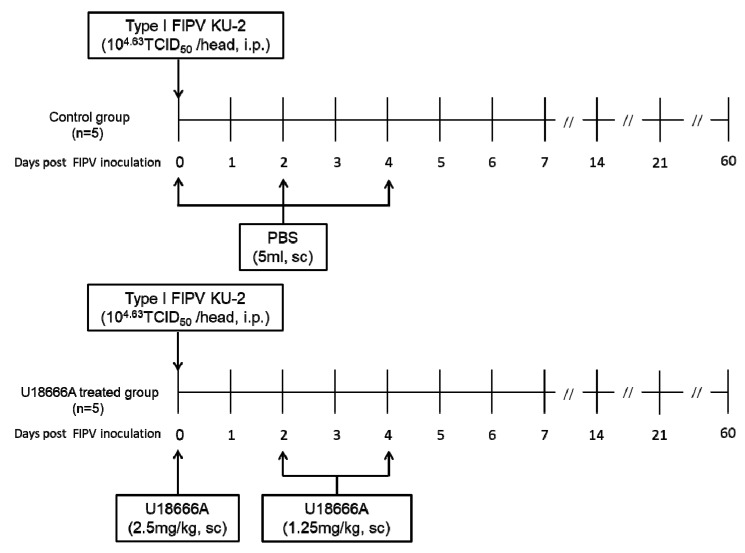
The experimental schedule of U18666A treatment and FIPV inoculation for cats.

The experimental schedule and information of cats are indicated in Figure 1 and Table 1, respectively. FIPV KU-2 (10^4.63^ TCID_50_/0.5 mL) was inoculated intraperitoneally to specific-pathogen-free (SPF) cats. The control group (n = 5) was administered PBS, and the U18666A-treated group (n = 5) was administered U18666A subcutaneously at 2.5 mg/kg on day 0, and 1.25 mg/kg on days 2 and 4 after viral inoculation. Cats were examined daily for clinical signs and their body temperatures and weights were measured. Blood was collected weekly using a heparinized syringe after the virus inoculation, and complete and differential cell counts were measured. In addition, saliva was collected every 2 days, feces were collected daily, and they were preserved until analysis at −80 °C. FIP diagnoses were confirmed upon postmortem examination, revealing peritoneal and pleural effusions, and pyogranuloma in the major organs. The cats were euthanized when reaching the humane endpoint or 63 days after the challenge.

### 2.2. Changes in Body Temperature and Body Weight

**Figure 2:**
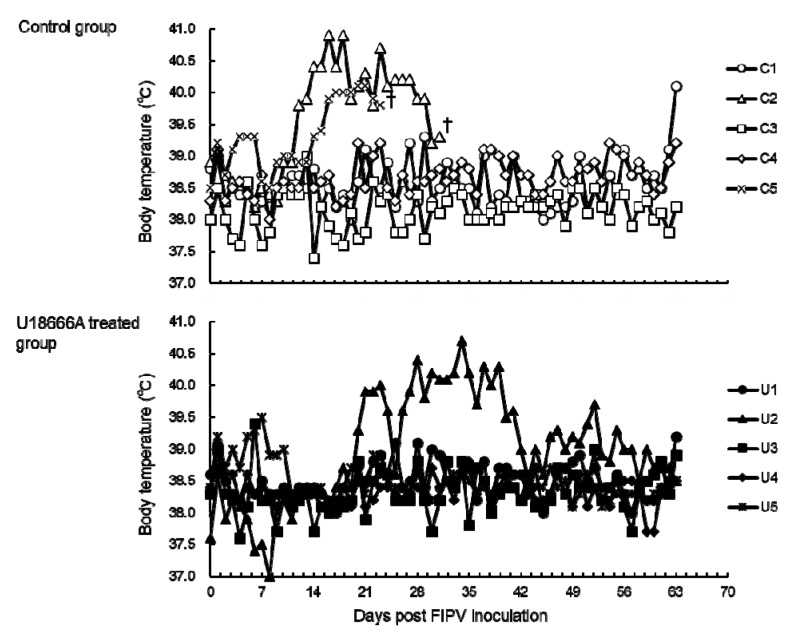
The changes in body temperature of FIPV-infected cats. †: The cat was euthanized because its clinical condition reached the humane endpoint.

The body temperature was measured serially in cats challenged with FIPV. In the control group, cats No. C2 and C5 developed fever above 40 °C from 14 days post-infection (d.p.i.) and 17 d.p.i., respectively (Figure 2), which persisted until the humane endpoint. In addition, cat No. C1 developed fever above 40 °C on 63 d.p.i. In the U18666A-treated group, cat No. U2 developed fever above 40 °C from 23 d.p.i. The fever persisted until 39 d.p.i., but began to decline on 40 d.p.i. and did not recur until the end of the experiment. No other cats exhibited fever above 40 °C throughout the experiment.

**Figure 3:**
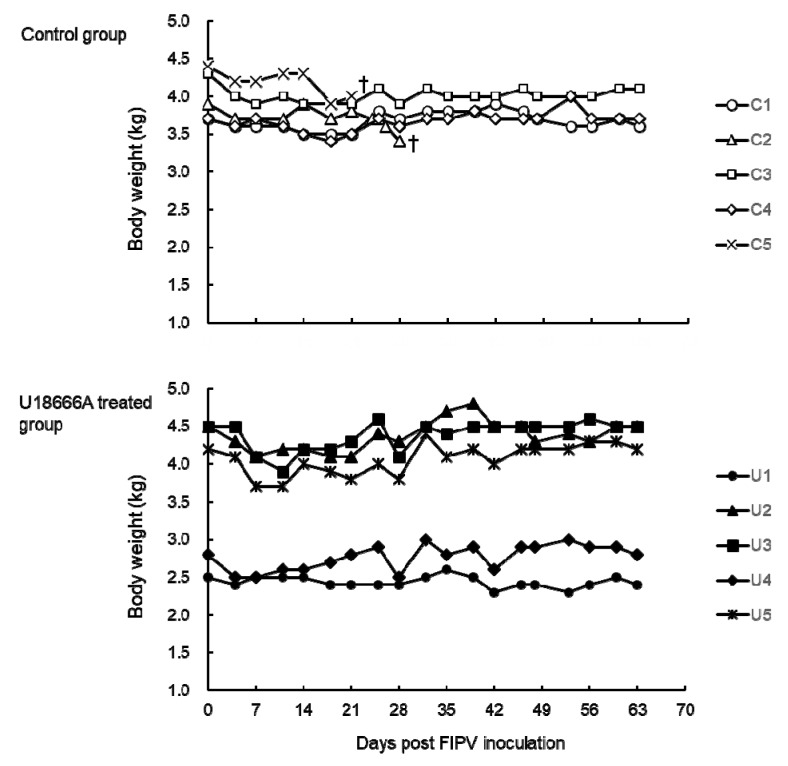
The changes in body weight of FIPV-infected cats. †: The cat was euthanized because its clinical condition reached the humane endpoint.

The body weight of the cats was measured after challenge with FIPV. In cats No. C2 and C5, which developed FIP, the body weight decreased from 1 week before the humane endpoint (Figure 3). Of the cats that did not develop FIP, the body weight of cat No. U3 decreased by 13% on 11 d.p.i. but increased thereafter and recovered at the end of the experiment to the level before viral challenge. In the other cats, little change in body weight was noted throughout the experiment.

### 2.3. The Changes on Anti-FCoV Antibody of FIPV-Infected Cats

**Figure 4:**
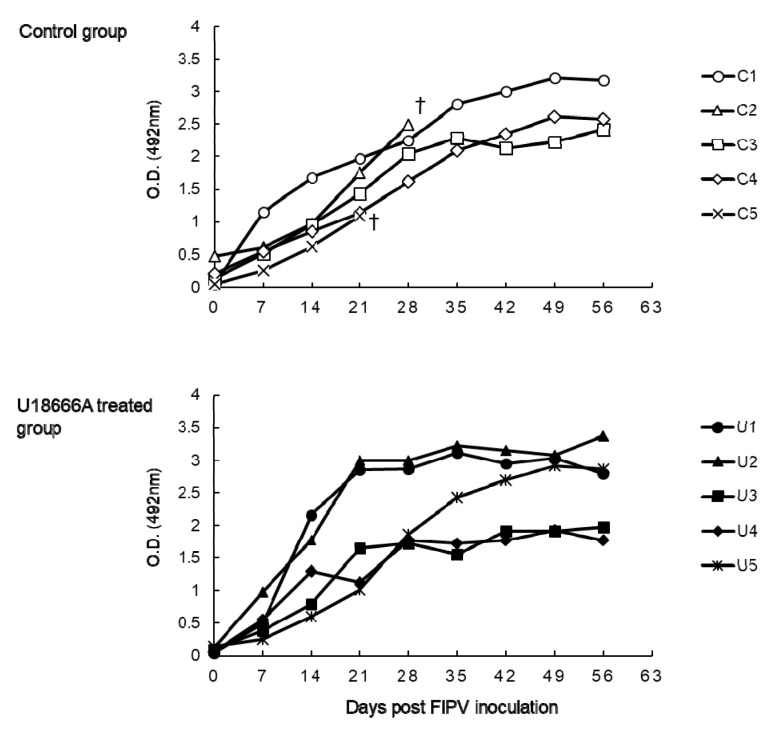
The changes on anti-FIPV antibody of FIPV-infected cats. †: The cat was euthanized because its clinical condition reached the humane endpoint.

Anti-FCoV antibody was assayed by ELISA using FIPV as the antigen (Figure 4). In all the cats, anti-FCoV antibody increased serially after 7 d.p.i.

### 2.4. Changes in the Peripheral Blood Lymphocyte Count

**Figure 5:**
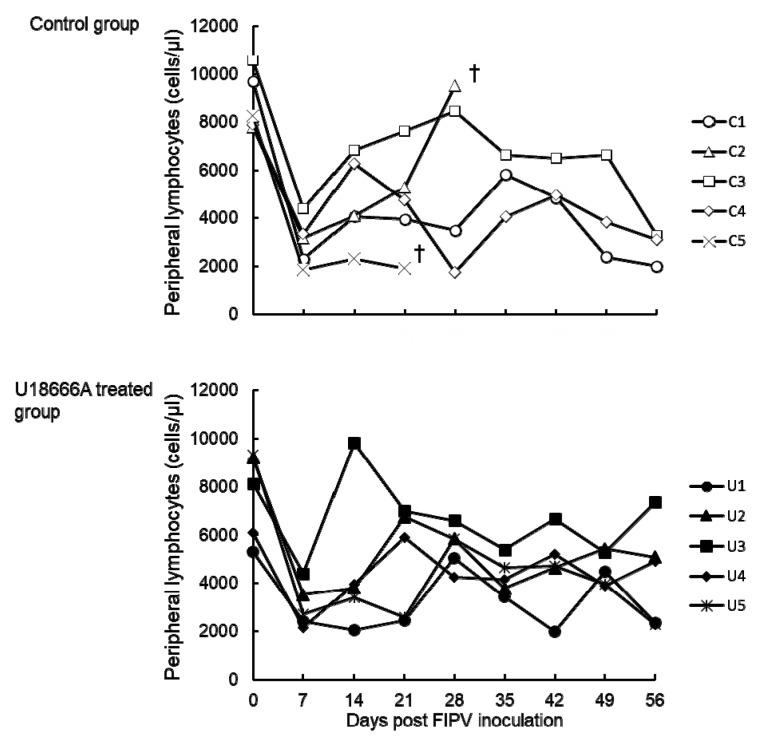
The changes on lymphocyte counts of FIPV-infected cats. †: The cat was euthanized because its clinical condition reached the humane endpoint.

The peripheral blood lymphocyte count was examined in cats challenged with FIPV. In all cats, the lymphocyte count decreased on 7 d.p.i. (Figure 5). After 14 d.p.i., the peripheral blood lymphocyte count in cat No. C5 remained low until the humane endpoint. In all the cats except cat No. C5, the peripheral blood lymphocyte count changed within the normal range (2000–8000 cells/μL in our laboratory).

### 2.5. Detection of the FIPV N Gene in Feces, Saliva, and Plasma Samples

**Figure 6:**
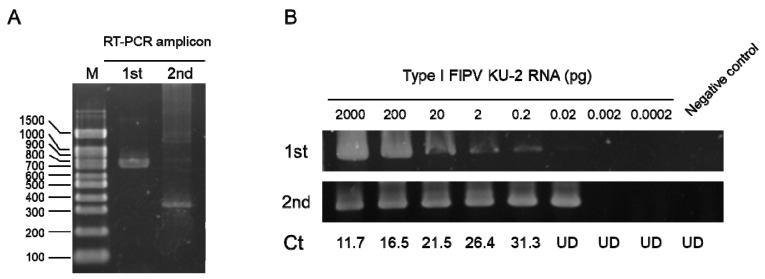
Sensitivity of nested-RT-PCR targeting the FIPV N gene. (**A**) RT-PCR amplicon. (**B**) Detection sensitivity of nested-RT-PCR for type I FIPV KU-2 RNA. Ct: Ct values by RT-qPCR targeting 3′-UTR. UD: Ct values were undefined.

To examine the viral shedding after inoculation of FIPV, nested-RT-PCR targeting the FIPV N gene was performed (Figure 6A). The sensitivity of nested-RT-PCR was assessed using purified type I FIPV KU-2 RNA. When compared with the Ct of quantitative real time RT-PCR (RT-qPCR) targeting 3′-UTR of FIPV reported by Kim et al., nested-RT-PCR demonstrated a higher detection sensitivity than RT-qPCR (Figure 6B) [16]. Based on these results, this nested-RT-PCR was used to detect the viral gene.

Viral shedding after intraperitoneally challenge with FIPV in feces, saliva and plasma was investigated. In the control group, the viral gene was detected in the feces samples of cats No. C2 (16–18 d.p.i.) and C3 (13–15, 16–18, and 28–30 d.p.i.) (Table 2). In the U18666A-treated group, the viral gene was detected in the feces samples of cats No. U1 (4–6 and 19–21 d.p.i.) and U5 (19–20 and 28–30 d.p.i.). In the control group, the viral gene was detected in the saliva samples of cats No. C1 and C5 at 10 d.p.i. and 8 d.p.i., respectively (Table 3). In the U18666A-treated group, the viral gene was detected in the saliva samples of cats No. U1 and U4 at 14 d.p.i. and 10 d.p.i., respectively. In cat No. U5, the viral gene was detected in the saliva samples at 6, 8, and 10 d.p.i. The viral gene was not detected in the plasma samples of any animal.

### 2.6. Survival Rate and Incidence of FIP

**Figure 7:**
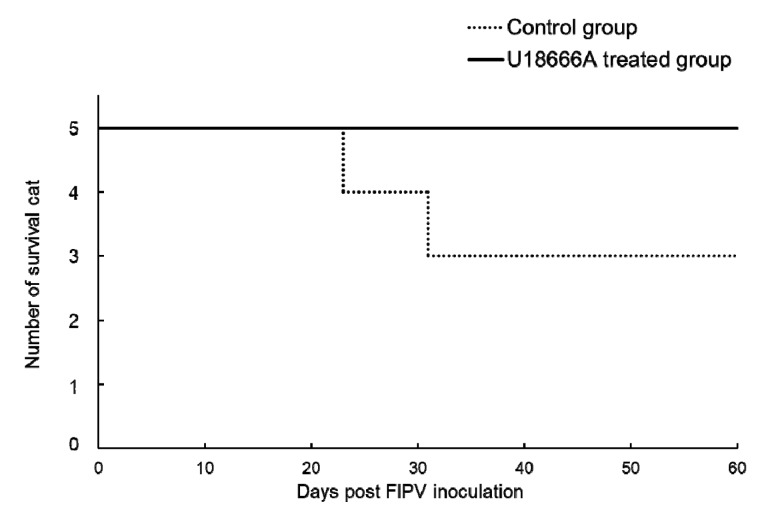
Survival rate of FIPV-infected cats. The cats were euthanized when reaching the humane endpoint.

The animals used for the experiment were euthanized on the day on which the humane endpoint was judged to have been reached and plotted on the survival curve (Figure 7). In the control group, cat No. C2 was euthanized at 23 d.p.i. and cat No. C5 was at 31 d.p.i., respectively. In the U18666A-treated group, none of the cats developed FIP and they all survived. The incidences of FIP in the control were 2 of 5 cats. In U18666A-treated groups, the incidences of FIP were 0 or suspected 1 of 5 cats.

### 2.7. Localization of Intracellular Cholesterol in Peripheral Blood Monocytes of SPF Cat Administered a Single Dose of U18666A

**Figure 8:**
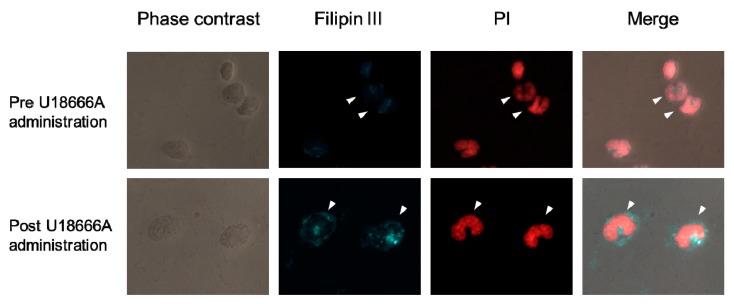
Localization of intracellular cholesterol in peripheral blood monocytes of SPF cat administered a single dose of U18666A. U18666A (2.5 mg/kg) was administered once to a SPF cat, and peripheral blood was collected before and 24 h after administration. Intracellular cholesterol of mononuclear cells, including peripheral blood monocytes, was stained by Filipin III, and the nucleus was stained by propidium iodide (PI). Arrowheads: peripheral blood monocytes.

One SPF cat was administered a single dose of U18666A. In monocytes of SPF cat before U18666A administration, intracellular cholesterol diffused over the entire cytoplasm (Figure 8). In monocytes of the same SPF cat 24 h after U18666A administration, cholesterol was found to be aggregated in the cytoplasm.

## 3. Discussion

Some viruses use cholesterol when infecting host cells. U18666A accumulates intracellular cholesterol by suppressing the function of the cholesterol transporter NPC1 [20]. The proliferation of DENV, HCV, ZIKV, and CHIKV is suppressed by U18666A [21,22,23,24]. We previously reported that U18666A suppresses the proliferation of type I FIPV by inhibiting the intracellular transport of cholesterol in a feline cell line [25]. However, the in vivo effects of U18666A remain unclear. In this study, we administered U18666A to cats, and evaluated its inhibitory effects on cholesterol transport and whether it has antiviral effects on FIPV.

There has been no report of the administration of U18666A to cats and its pharmacokinetics in cats are unknown. In rats, however, the pharmacokinetics of U18666A after administration were previously investigated [26]. U18666A is distributed in the brain from immediately after subcutaneous or intraperitoneal administration. In rats, U18666A demonstrated a biphasic elimination pattern consisting of early-rapid clearance (T1/2 = 16 h) 24–36 h after administration and subsequent slow and sustained clearance (T1/2 = 58 h). Based on this report, we assumed the distribution volume in cats to be 600 mL/kg and set a single dosage necessary to obtain an initial blood concentration of 10 μM (2.5 mg/kg: 6 μM/kg). In cats administered U18666A at this dose, the blood concentration was estimated to reach 2 μM or above within 24 h after administration. This concentration is 10 times higher than the EC_50_ of U18666A against type I FIPV KU-2 determined in an in vitro experiment (0.2 μM) [27].

We administered U18666A to a SPF cat once and examined whether intracellular cholesterol aggregated in peripheral blood monocytes. In the monocytes of SPF cat before U18666A administration, intracellular cholesterol diffused over the entire cytoplasm. In monocytes of the same SPF cat 24 h after U18666A administration, cholesterol was found to be aggregated in the cytoplasm. In monocytes of SPF cats administered U18666A, intracellular cholesterol accumulation was induced.

The in vivo antiviral effects of U18666A were examined by administering U18666A to SPF cats experimentally infected with type I FIPV. After challenge with type I FIPV KU-2, U18666A was administered at 2.5 mg/kg once and 1.25 mg/kg twice thereafter in 48-h intervals. No epileptic symptoms were noted in the cats administered U18666A a total of three times. Therefore, the administration method of U18666A employed in this study was suggested to be safe in cats. However, in consideration of studies of other drugs for FIP, it is necessary to administer U18666A for a long period until FIP remission. U18666A, which is amphiphilic, is likely to accumulate in the brain and adipose tissue. Indeed, epileptic symptoms were reported to be induced in rats by the repetitive administration of U18666A at a high dose (10 mg/kg) [26]. To avoid such adverse effects, it is necessary to investigate the pharmacokinetics of U18666A in cats in greater detail.

One week after viral inoculation, the peripheral blood lymphocyte count decreased and anti-FCoV antibody increased in all the cats. Of the five control cats administered PBS alone, two exhibited persistent fever, body weight loss, and loss of appetite, and subsequently developed FIP. These two cats had no improvement in FIP symptoms and were euthanized. However, of the five cats administered U18666A, four developed no signs of FIP. One cat that temporarily developed fever had no other clinical symptoms, and no gross lesion was noted on autopsy after the end of the experiment. The incidences of FIP in the control were two of five cats. In U18666A-treated groups, the incidences of FIP were zero or suspected to be one of five cats. In a previous experiment, FIPV KU-2 caused FIP at a rate of 20%–40% by intraperitoneal inoculation [28,29]. On the bases on those facts, U18666A might have suppressed the development of FIP in FIPV-infected cats. However, the number of animals with FIP is too low to establish anti-viral effect of U18666A in cats. Further, compared with effective anti-viral drugs such as GS-441524 and GC-376, U18666A seems to have a weak anti-viral effect in vivo.

After viral inoculation, samples serially collected from the control and U18666A-treated groups were analyzed to detect the FIPV N gene. The FIPV N gene was detected intermittently in feces and saliva regardless of the development of FIP or administration of U18666A. Pedersen et al. reported that viral genes are rarely detected in tissues not directly involved in viral proliferation such as feces or blood. They also suggested that whether the viral gene is detected is unrelated to the pathological manifestation of FIP [30]. These observations suggest that it is difficult to evaluate the in vivo proliferation of type I FIPV in cats using feces, saliva and plasma samples, although they can be collected continuously by minimal or non-invasive procedures.

According to Pedersen et al., viral genes are readily detected in tissues closely related to viral proliferation (omentum, mesenteric lymph nodes, and spleen) and effusion [30]. Among effusion types, peritoneal effusion, in particular, can be collected relatively easily. Indeed, some compounds with antiproliferative effects on FIPV have been confirmed to have antiviral effects based on the measurement of the amount of viral gene in peritoneal effusion of cats that developed FIP. However, the use of such an evaluation method is limited to cats that have developed FIP and have peritoneal effusion. In the future, development of an exact and simple method for the measurement of the amount of virus is anticipated.

U18666A was suggested to suppress the proliferation of type I FIPV KU-2 in vivo and reduce the incidence of FIP, i.e., it is promising as an anti-FIPV drug. We previously reported Itraconazole as a drug that suppresses the proliferation of type I FIPV by inhibiting the intracellular cholesterol transport similarly to U18666A [25,27]. As Itraconazole is used in clinical veterinary practice as an antifungal agent; it can be promptly applied to cats. Furthermore, the SI value (CC_50_/EC_50_) of U18666A is 2.8-times higher than that of Itraconazole. This suggests that U18666A is clinically safer than Itraconazole as an anti-FIPV drug. In addition, antiviral agents with high blood-brain barrier penetrability may be necessary to improve the condition of FIP cats exhibiting central nervous system symptoms. U18666A, which is highly transferable to the brain, may also be effective for the management of neurological symptoms of FIP. From these observations, U18666A, along with Itraconazole, is considered to be a potential antiviral agent applicable to type I FIPV.

In this study, we administered U18666A to cats and evaluated its inhibitory effects against cholesterol transport and antiviral effects against FIPV. U18666A administered to cats induced cholesterol accumulation in the cytoplasm of peripheral blood monocytes. When U18666A was administered to cats experimentally infected with type I FIPV, the development of FIP might be suppressed compared with the control group. However, the number of animals with FIP is too low to establish anti-viral effect of U18666A in cats. In the future, it is necessary to examine the pharmacokinetics of U18666A in cats and evaluate its therapeutic effects in cats diagnosed with FIP.

## 4. Materials and Methods

### 4.1. Virus

Type I FIPV KU-2 was grown in Felis catus whole fetus (fcwf)-4 cells at 37 °C. The type I FIPV KU-2 was isolated in our laboratory.

### 4.2. U18666A

U18666A was purchased from Sigma Aldrich (St. Luis, MO, USA). U18666A was dissolved in phosphate-buffered saline (PBS) (pH 7.4) at 10 mg/mL and sterilized by filtering through a 0.20 μm filter.

### 4.3. Animal Experiments

All applicable national and institutional guidelines for the care and use of animals were followed. The animal experimentation protocol was approved by the President of Kitasato University through the judgment of the Institutional Animal Care and Use Committee of Kitasato University (approval No. 17-062). The SPF cats were bred in our own laboratory and maintained in a temperature-controlled isolated facility.

### 4.4. ELISA

The ELISA for anti-FCoV antibodies was performed as described by Takano et al. [29]. Briefly, detergent-disrupted, purified FIPV virions were diluted appropriately with carbonate buffer (0.05 M, pH 9.6). A total of 100 μL of the dilution was pipetted into each well of a 96-well flat-bottomed plate. The plates were allowed to stand overnight at 4 °C, washed with PBS containing 0.02% Tween-20, and 100 μL of the test plasma sample was then added to each well. Horseradish peroxidase-conjugated goat anti-cat IgG (ICN Pharmaceuticals Inc., Costa Mesa, CA, USA) was diluted to the optimal concentration with PBS containing 10% FCS and 0.05% Tween-20, and 100 μL of dilution was added to each well of plates. After incubation at 37 °C for 30 min, 100 μL of the substrate solution was added to each well and plates were incubated at 25 °C for 20 min in a dark room. The substrate solution was prepared by dissolving o-phenylenediamine dihydrochloride at a concentration of 0.4 mg/mL in 0.1 M citric acid and 0.2 M Na_2_HPO_4_ buffer (pH 4.8), and 0.2 μL/mL of 30% H_2_O_2_ was then added. The reaction was stopped with 3 N H_2_SO_4_ solution and the optical density (O.D.) at 492 nm was determined.

### 4.5. RNA Isolation and cDNA Preparation

Total cellular RNA was extracted from feces, saliva and plasma using a High Pure RNA Isolation Kit (Roche Diagnostics GmbH, Mannheim, Germany) according to the instructions of the manufacturer. RNA was dissolved in elution buffer. Using total cellular RNA as a template, cDNA was synthesized using PrimeScript™ 1st strand cDNA Synthesis Kit (Takara Bio Inc., Kusatsu, Japan). Reverse transcription was performed in a 20 μL final volume containing 1 μL of oligo (dT) primer (50 μM). The resulting solution was incubated at 42 °C for 30 min to synthesize cDNA.

### 4.6. Detection of Feline FIPV N Gene

cDNA was amplified by nested-PCR using specific primers for feline FIPV N gene. The primer sequences are shown in Table 4. PCR was performed in a total volume of 50 μL. One microliter of sample cDNA was mixed with 25 μL of Quick Taq HS DyeMix (TOYOBO Co., Osaka, Japan), 2 μL of 10 μM primer mix, and 22 μL of distilled water. Using a PCR Thermal Cycler Dice (Takara Bio Inc., Kusatsu, Japan), the DNA was amplified at 94 °C for 3 min, followed by 30 cycles of denaturation at 94 °C for 1 min, primer annealing at 55 °C for 1 min, and synthesis at 72 °C for 2 min, with a final extension at 72 °C for 5 min. The 1st and 2nd rounds of PCR were performed by the same thermal protocol. The PCR products were resolved by electrophoresis on 3% agarose gels. The gels were incubated with ethidium bromide solution (1 μg/mL), and bands were visualized using a UV transilluminator at 312 nm and photographed.

To compare the sensitivity of nested-RT-PCR, RT-qPCR was performed using a primer targeting 3′-UTR reported by Kim et al. [16]. Briefly, RNA was reverse-transcribed and amplified using RNA-direct Realtime PCR Master Mix (TOYOBO Co., Osaka, Japan) with the specific primers 3′-UTR-F (5′-GGAGGTACAAGCAACCCTATT-3′) and 3′-UTR-R (5′-GATCCAGACGTTAGCTCTTCC-3′) and a probe (FAM-5′-AGATCCGCTATGACGAGCCAACAA-3′-BHQ1). The reaction was carried out in a total volume of 20 μL/well in 48-well PCR plates using a StepOne Real-Time PCR System (Thermo Fisher Scientific, Waltham, MA, USA) at 90 °C for 30 s, 60 °C for 20 min, and 95 °C for 1 min, followed by 45 cycles of 90 °C for 15 s and 60 °C for 1 min. Nested-RT-PCR and RT-qPCR were performed, and their sensitivities were compared. As a template, RNA purified from the culture supernatant of type I FIPV KU-2 was used.

### 4.7. Single Administration of U18666A and Staining of Cellular Cholesterol in Monocytes

A cat was administered U18666A subcutaneously at 2.5 mg/kg. Peripheral blood was collected before and 24 h after administration. Heparinized blood was 2-fold diluted with PBS, and subjected to Ficoll-Hypaque (lymphoprep, Alere Technologies AS, Oslo, Norway) density gradient centrifugation at 800 g for 20 min. The PBMC layer was collected and washed three times. After cell counting, the PBMC was seeded to an 8-well Lab-Tek Chamber Slide (Thermo Fisher Scientific, Waltham, MA, USA) at density of 1 × 10^5^ cells/well. Slide was incubated at 37 °C for 2 h. After washing three times, adherent cells were fixed and stained. The cellular cholesterol was stained by the Cholesterol Cell Based Detection Assay Kit (Cayman chemical, Ann Arbor, MI, USA) according to the manufacturer’s instructions. After filipin III staining, cells were stained by propidium iodide (PI, NACALAI TESQUE, INC., Kyoto, Japan) and analyzed using a Leica DM4B microscope with LAS X integrated imaging system (Leica Microsystems, Wetzlar, Germany).

## Figures and Tables

**Table 1:** Information of cats.

	Cat No.	Gender	Age (Months)
Control group	C1	Female	13
	C2	Male	13
	C3	Male	13
	C4	Male	12
	C5	Female	8
U18666A treated group	U1	Female	13
	U2	Male	13
	U3	Male	12
	U4	Female	12
	U5	Male	8

**Table 2:** Detection of FIPV N gene from feces of FIPV-infected cats.

		Days Post FIPV Inoculation										
	Cat No.	0	1–3	4–6	7–9	10–12	13–15	16–18	19–21	22–24	25–27	28–30
Control group	C1	−	−	−	−	−	−	−	−	−	−	−
	C2	−	−	−	−	−	−	+	−	−	−	−
	C3	−	−	−	−	−	+	+	−	−	−	+
	C4	−	−	−	−	−	−	−	−	−	−	−
	C5	−	−	−	−	−	−	−	−	N.D.	N.D.	N.D.
U18666A treated group	U1	−	−	+	−	−	−	−	+	−	−	−
	U2	−	−	−	−	−	−	−	−	−	−	−
	U3	−	−	−	−	−	−	−	−	−	−	−
	U4	−	−	−	−	−	−	−	−	−	−	−
	U5	−	−	−	−	−	−	−	+	−	−	+

N.D.: not done.

**Table 3:** Detection of FIPV N gene from saliva of FIPV-infected cats.

		Days Post FIPV Inoculation						
	Cat No.	0	2	4	6	8	10	14
Control group	C1	−	−	−	−	−	+	−
	C2	−	−	−	−	−	−	−
	C3	−	−	−	−	−	−	−
	C4	−	−	−	−	−	−	−
	C5	−	−	−	−	+	−	−
U18666A treated group	U1	−	−	−	−	−	−	+
	U2	−	−	−	−	−	−	−
	U3	−	−	−	−	−	−	−
	U4	−	−	−	−	−	+	−
	U5	−	−	−	+	+	−	−

**Table 4:** Sequence of nested-RT-PCR Primers.

	Orientation	Nucleotide Sequence	Location	Length (bp)	Reference
1st	Forward	5′-CAACTGGGGAGATGAACCTT-3′	23–42	788	KC461235
	Reverse	5′-GGTAGCATTTGGCAGCGTTA-3′	791–810		
2nd	Forward	5′-ATTGATGGAGTCTTCTGGGTTG-3′	324–345	362	KC461235
	Reverse	5′-TTGGCATTCTTAGGTGTTGTGTC-3′	663–685		
